# Treatment of Locally Advanced Melanoma by Isolated Limb Infusion with Cytotoxic Drugs

**DOI:** 10.1155/2011/106573

**Published:** 2011-07-21

**Authors:** Hidde M. Kroon

**Affiliations:** ^1^Melanoma Institute Australia, Royal Prince Alfred Hospital, University of Sydney, Missenden Road Camperdown, NSW 2050, Australia; ^2^Rijnland Hospital, Simon Smithweg 1, 2353 GA Leiderdorp, The Netherlands

## Abstract

Since its introduction in the late 1950s, isolated limb perfusion (ILP) has been the preferred treatment option for locally advanced melanoma and sarcoma confined to a limb. This treatment results in high response rates with a satisfying duration of response in both tumor types. A drawback of ILP, however, is the invasive and complex character of the procedure. Isolated limb infusion (ILI) has been designed in the early 1990s as a minimally invasive alternative to ILP. Results of this simple procedure, reported by various centers around the world, show comparable response rates for melanoma and sarcoma when compared to ILP. Due to its minimally invasive character, ILI may replace ILP in the future as the preferred treatment for these locally advanced limb tumors.

## 1. Introduction

Patients who suffer from advanced melanoma or sarcoma confined to a limb are often challenging to treat due to the size and number of the satellite and/or in-transit metastasis in melanoma or the invasion of the tumor in sarcoma. In the past, results of systemic therapies in these tumours have often been disappointing. Although promising results have been published using ipilimumab and RG 7204 for metastatic melanoma, little is known about the effect of these agents when metastases are limited to a limb [1–4]. In the past an amputation was often inevitable but since the late 1950s this mutilating procedure can be avoided in the majority of patients by performing an isolated limb perfusion (ILP). 

During ILP the blood circulation of the limb is temporarily discontinued from the systemic circulation. With a surgical procedure the femoral artery and vein (when treating a leg) or the axillary artery and vein (when treating an arm) are clamped and connected to an extracorporeal circuit containing a heart-lung machine in order to preserve physiological circumstances in the isolated limb. To achieve optimal isolation, minor vessels of subcutaneous tissues and muscles are compressed by a tourniquet. In this isolated circuit the dose of the cytotoxic drug, normally melphalan, can be safely applied up to a tenfold higher than systemically tolerated without compromising locally irreversible adverse effects [4–6]. Since flow of the cytotoxic drug to the systemic circulation could result in a life-threatening situation, potential leakage is continuously monitored during the procedure [7]. The cytotoxic drug circulates typically 60 to 90 minutes after which the limb is flushed to discard the remaining drugs in the isolated limb. The procedure is finalized by surgically disconnecting the tubes of the heart-lung machine, closing the vessels with sutures or a patch and deflating the tourniquet to restore the normal circulation in the limb. 

Following ILP for melanoma complete response (CR) percentages of 7–91 (median 46) and partial response (PR) percentages of 0–44 (median 34) are reported. The median recurrence-free survival is 14 months, which is 23 months (range 8– > 72) following a CR. Overall survival following ILP is 24 months and 44 months (IQR 22– > 120) after a CR [4, 8, 9]. In patients with locally advanced sarcoma response percentages of 63–91 are reported [5, 10]. In both patient groups ILP can prevent amputation of the affected limb in 90% of the cases. 

Despite these excellent results, ILP has some major disadvantages. It is an invasive and technically complex procedure in which not only a surgeon is involved but also a perfusionist and a large number of supporting staff are needed [11]. Although some have reported that ILP can be performed safely in elderly and frail patients, it is only performed in carefully selected patients [12–14]. Finally, a repeat ILP, after disease recurrence, is complex and can result in major complications due to scar tissue from the previous surgical approach of the vessels.

## 2. Isolated Limb Infusion

In the early 1990s the isolated limb infusion (ILI) technique was developed by Thompson and colleagues at the Sydney Melanoma Unit (currently Melanoma Institute Australia; MIA) as a simplified and minimally invasive alternative to ILP [25]. In contrast to ILP, during ILI no invasive surgical approach is needed. Radiological catheters with additional side holes near their tips are inserted percutaneously into the axial artery (6 French) and vein (8 French) of the disease-bearing limb via the contralateral groin using the Seldinger technique. Their tips are positioned in such a way that they are at the level of the knee or elbow joint. Tissues more proximally located in the limb but distal to the level of the tourniquet were perfused in a retrograde fashion via collateral vascular channels. The patient is then given a general anesthetic, and heparin (3 mg/kg) is infused to achieve full systemic heparinization. The catheters are connected to an extracorporeal circuit filled with saline solution incorporating a blood-warming coil but without a heart-lung machine. A pneumatic tourniquet is inflated around the root of the to be treated limb, and the cytotoxic agents are infused into the isolated circuit via the arterial catheter. In this isolated circuit a low-flow circulation can be realized without oxygenating the circulated blood resulting in a hypoxic and acidotic environment (Table 1) [17]. The cytotoxic drugs that are used are melphalan 5–10 mg/L of tissue (mostly 7.5 mg/L) and actinomycin-D 50–100 *μ*g/L of tissue (mostly 75 *μ*g/L) in 400 mL warmed, heparinized normal saline. Actinomycin-D is used because of the good response rates (CR 73%) of the melphalan/actinomycin-D combination when administered by conventional ILP, without excessive limb toxicity [26].

After infusion of the drugs in the isolated circuit the infusate is continually circulated for 30 minutes by repeated aspiration from the venous catheter and reinjection into the arterial catheter using a syringe attached to a three-way tap in the external circuit. 

Since the half time of melphalan is 15 to 20 minutes and both melphalan and actinomycin-D are quickly absorbed by the tissues of the isolated limb, a relative short circulation time of 30 minutes is sufficient [27, 28]. 

Great care is given to the limb temperature since cooling of the extremity has a negative effect on the efficacy of the cytotoxic drugs. Heating of the limb is achieved by the aforementioned blood-warming coil in the extracorporeal circuit and by encasing the limb in a hot-air blanket, with a radiant heater placed over it [6]. Subcutaneous and intramuscular limb temperatures are monitored continuously during the ILI procedure. If these precautions are taken into account it is possible to achieve limb temperatures just above 40°C. Blood samples are taken at regular intervals to measure the melphalan concentrations and blood gases (Table 1). The drug leakage rate from the isolated limb is assessed retrospectively on the basis of systemic melphalan concentrations that are measured routinely during each procedure. Intraoperative systemic leakage monitoring is not performed because systemic leakage is negligibly low due to the low-flow and low-pressure circuit of the isolated limb and the effective isolation using the tourniquet. 

**Figure 1:**
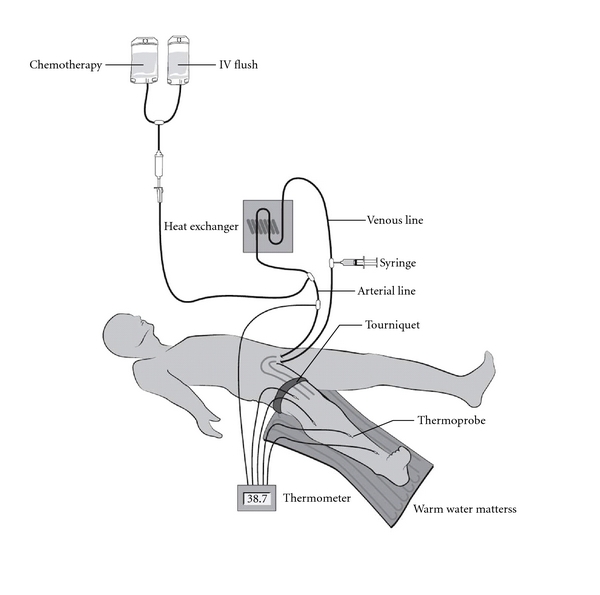
Schematic illustration of the circuit used for isolated infusion of a lower limb [15].

**Figure 2:**
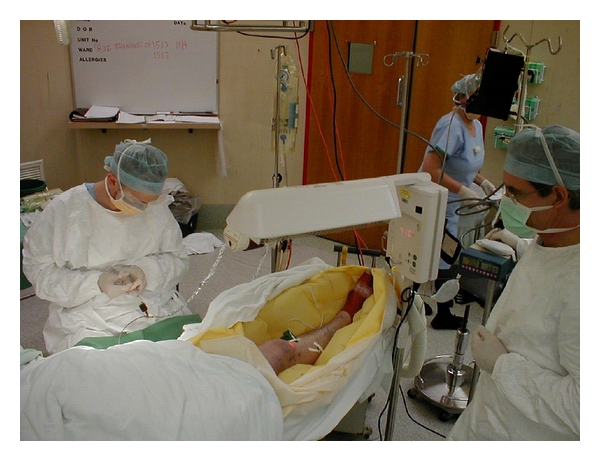
Photograph of an isolated limb infusion procedure in progress in the operating theatre [16].

After 30 minutes, the limb is flushed with one liter of Hartmann's solution via the arterial catheter, and the venous effluent was discarded. The tourniquet is then deflated to restore normal limb circulation, the heparin is reversed with protamine, and the catheters are removed [29]. Figures 1 and 2 provide an overview of the procedure [15, 16].

Postoperatively the serum creatine phosphokinase (CK) level is measured daily as an indicator for muscle and tissue breakdown, and limb toxicity, systemic toxicity, and tumor response are assessed regularly.

## 3. Results of Isolated Limb Infusion

**Figure 3:**
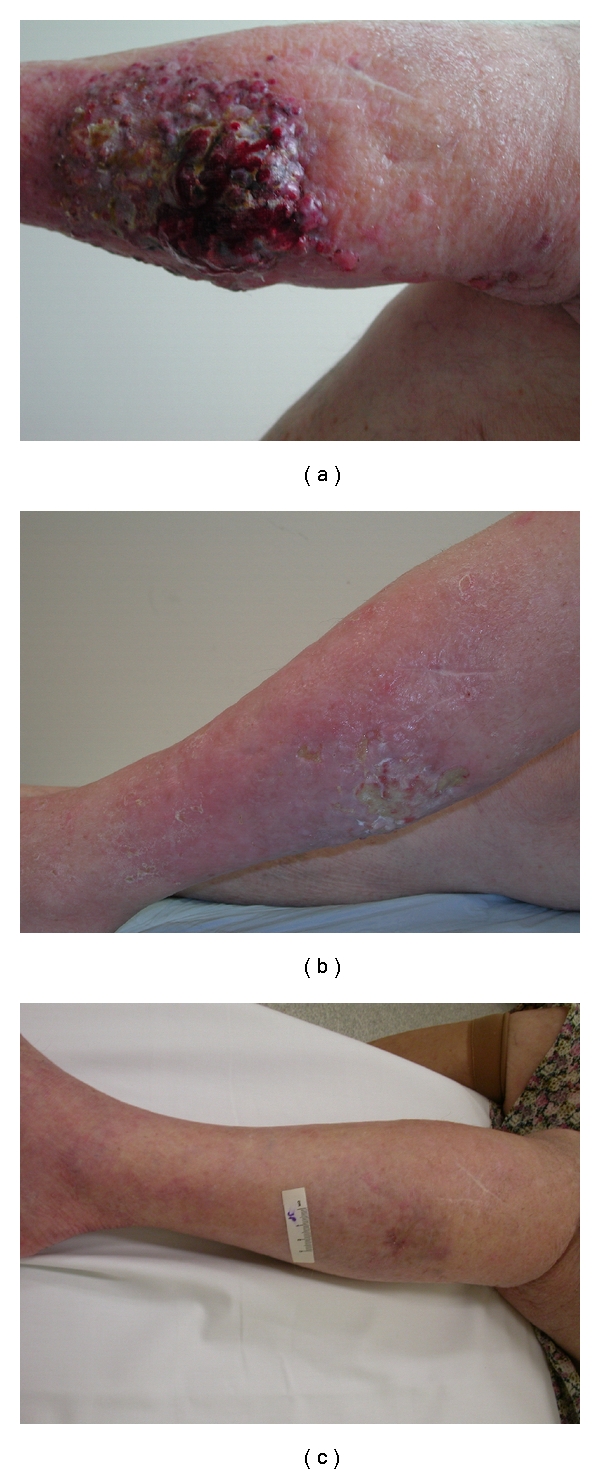
(a) Extensive in-transit melanoma metastases of the left lower leg before ILI. (b) Remission 4 weeks post-ILI. (c) Complete response 4 months post-ILI.

Since 1992 over 400 ILIs have been performed in the MIA, mostly for melanoma but also for patients with locally advanced sarcoma. Following ILI a CR rate of 38% and a PR of 46% are seen in patients suffering from melanoma (Figure 3) [17]. The median LRFI in these patients was 13 months and 22 months (range 5 to >72: *P* = .012) for those experiencing a CR. The median survival following a CR was 53 months (range 28 to >120), 26 months (range 14 to >120) following a PR, and only 6 months for the small group of patients who had stable or progressive disease following the procedure (*P* = .004). These results are comparable to those reported after ILP [30, 31]. To date only one multicenter retrospective analysis for ILI has been published [18]. In this analysis 31% of the patients experienced a CR, 33% a PR, and 36% showed no response to the treatment. In addition to these institutions a number of institutions around the world have reported their experiences. These are listed in Table 2 [18–22]. The wide range of the results in these studies is possibly caused by the low number of patients in some of the studies and possibly by the lack of experience with this new technique. Furthermore, all institutes have used a protocol that is different in very small, but potentially essential, ways. The impact of these differences in protocol and the effect of increased experience have recently been investigated by Huismans et al. [32]. They showed that increased experience and small modifications that were made to the ILI protocol at the MIA over the years resulted in a positive effect on the outcome. Another explanation for the reported range in results could be the point in time when the response of the procedure was investigated. Beasley et al., for instance, took the response after exactly 3 months while others took the best response at any time after ILI [20]. Despite the differences in experience, protocol, and outcome, most investigators have reported that patients who obtain a CR have significantly improved survival compared with nonresponders [20, 22].

The experience in using ILI for inoperable sarcoma is still limited, to two studies. However, the results reported are comparable to those seen after ILP [16, 23]. In these two separate studies CR rates of 23 and 57% and PR rates of 27 and 33% were reported with a median LRFI of 15 months. Following ILI amputation of the affected limb could be avoided in 76–94% of the sarcoma patients. 

Following ILI the regional toxicity due to the cytotoxic drug is low [20, 25, 33]. Slight erythema and oedema are seen in 41–57% of the patients, and in 39–53% this is accompanied with the forming of blisters. In most cases a conservative treatment involving bed rest, elevation, and sometimes administering steroids is sufficient. In 3% of the patients the deeper tissues are involved, and in order to prevent a compartment syndrome a fasciotomy is sometimes carried out. To date at the MIA, it has not been necessary to amputate a limb due to severe tissue damage following ILI with melphalan and actinomycin-D. A study focusing on toxicity showed that patients with larger limb volumes experience increased toxicity grades without receiving higher cytotoxic drug doses [34]. In order to decrease the toxicity rates in these patients Beasley et al. corrected the melphalan dose for ideal body weight (IBW) [35]. In their hands this decreased toxicity significantly (*P* = .001) with only a small insignificant decrease in response (*P* = .345). However, these results could not be reproduced in a study initiated by the MIA in which the ratio of IBW and actual body weight did not predict toxicity or outcome (unpublished data) [36].

## 4. Discussion

One of the main advantages of ILI is the minimally invasive character of the procedure. 

Morbidity as a result of the surgical approach of the blood vessels as seen in ILP is not experienced, and normally patients can be discharged from the hospital 7 days after the procedure [32, 34]. ILI can also safely be performed in elderly and frail patients without risking severe adverse effects. No increase in toxicity or morbidity was seen in the MIA patients, despite the fact that their average age was considerably higher than those seen in most ILP studies [17, 37]. Even in patients who suffer from distant metastatic disease and concurrent symptomatic limb disease ILI can effectively be used as a palliative treatment to provide local tumor control and limb salvage [38]. Also, because scar tissue is hardly formed following ILI, the procedure can easily be repeated in case of recurrent disease. The response rates of these repeat procedures are comparable to those seen after a first ILI [39].

Another advantage of ILI is the hypoxemia and acidosis in the isolated limb. Animal studies have shown that a hypoxic and acidotic environment enhances the effect of melphalan by a factor 3 [40]. Clinically enhanced responses were observed when isolation of the limb lasted longer than 40 minutes [21].

Furthermore, ILI has a number of practical advantages over ILP. The time in the operating theatre is considerably shorter (on average one hour compared to 3–5 hours for ILP), no complex and expensive equipment is used, and less personnel is needed [29]. Because of this, ILI is a much cheaper procedure. Finally, ILI is often used in trial settings to provide insight for developing novel treatment strategies [41]. One of these studies used systemic ADH-1 in combination with melphalan. It was well tolerated and provided a CR of 50% [42]. In Table 3 differences between ILI and ILP are listed.

## 5. Conclusion

Over the last two decades ILI has become a serious alternative to the traditionally used ILP technique. Studies, most published in the recent years, have shown that, when performed correctly, response rates following ILI are comparable to those seen after ILP. ILI, however, results in less toxicity and morbidity. In the future more research and in particular a randomized controlled trial is needed to prove the effect of ILI, and it is not unthinkable that ILI will become the preferred treatment for patients who suffer from advanced melanoma or sarcoma confined to a limb.

## Figures and Tables

**Table 1:** Mean blood gas values of the isolated limb after 30 minutes in 185 patients [17].

pO_2_	8.4 mmHg
pCO_2_	54.3 mmHg
pH	7.11
BE	−10.8 mmol/L
SO_2_	6.9%

**Table 2:** Isolated limb infusion studies using melphalan and actinomyocin-D [17–23].

Author, year	No. of patients	Response criteria	CR	PR	SD	PD
Mian et al., 2001 [19]	9*	Best response	44%	56%	0%	0%
Lindnér et al., 2002 [21]	128	Best response	41%	43%	12%	4%
Brady et al., 2006 [23]	22**	3 months	23%	27%	0%	50%
Kroon et al., 2008 [17]	185	Best response	38%	46%	10%	6%
Beasley et al., 2008 [20]	50	3 months	30%	14%	10%	46%
Marsden, 2008 [24]	16***	Unknown	26%	58%	—	16%
Barbour et al., 2009 [22]	74	Best response	24%	30%	37%	7%
Beasley et al., 2009 [18]	128	3 months	31%	33%	7%	29%

CR: complete response; PR: partial response; SD: stable disease; PD: progressive disease.

*3 patients had >1 ILIs.

**1 patient had advanced sarcoma.

***3 patients had >1 ILIs, 4 patients had squamous cell carcinoma, and 2 patients had Merkel cell carcinoma.

**Table 3:** Differences between isolated limb perfusion and isolated limb infusion.

Isolated limb perfusion	Isolated limb infusion
Technically complex	Technically simple
Open surgical exposure of vessels for catheter insertion	Percutaneous vascular catheter insertion in radiology department
4 to 6 hours duration	Approximately 1 hour
Perfusionist and large number of staff required	No perfusionist required and fewer total staff
Complex and expensive equipment needed	Equipment requirements modest
Magnitude of procedure excludes patients	Well tolerated by medically compromised, frail, and elderly patients
Not possible in occlusive vascular disease	Can be performed in occlusive vascular disease
Technically challenging to perform a repeat procedure	Not difficult to perform a repeat procedure
Systemic metastases normally a contraindication	Systemic metastases not a contraindication
Higher perfusion pressures predispose to systemic leakage	Low pressure system, effective vascular isolation with tourniquet
Limb tissues oxygenated, with normal blood gases maintained	Progressive hypoxia and acidosis
Hyperthermia (>41°C can be achieved)	Usually not possible to raise limb temperature above 40°C
General anesthesia required	Possible with regional anesthesia
